# Effect of home-based pulmonary rehabilitation on exercise capacity in post COVID-19 patients: a randomized controlled trail

**DOI:** 10.1186/s12984-024-01340-x

**Published:** 2024-03-25

**Authors:** Tamer I. Abo Elyazed, Laila A. Alsharawy, Shaimaa E. Salem, Nesma A. Helmy, Ahmed Abd El-Moneim Abd El-Hakim

**Affiliations:** 1 Department of Physical Therapy for Internal Medicine, Faculty of Physical Therapy, Beni-Suef University, Beni Suef, Egypt; 2 Department of Chest Disease, Faculty of Medicine, Beni-Suef University, Beni Suef, Egypt; 3 Department of Basic Sciences, Faculty of Physical Therapy, Cairo University, Giza, Egypt; 4 Department of Basic Sciences, Faculty of Physical Therapy, Al Ryada University for Science and Technology, Sadat City, Egypt; 5 Department of Basic Sciences, Faculty of Physical Therapy, Beni-Suef University, Beni Suef, Egypt

**Keywords:** Post COVID-19, Pulmonary rehabilitation, Functional capacity

## Abstract

**Background:**

Coronavirus 2019 (COVID-19) is an epidemic condition that compromises various consequences. The goal of this study was to investigate the effect of home-based pulmonary rehabilitation on exercise capacity in patients with post COVID-19 syndrome.

**Methods:**

The study was designed as a randomized control trial. A total of sixty-eight patients with post COVID-19 syndrome complaining of fatigue, dyspnea, and exercise intolerance participated in this study. Their ages ranged from 40 to 70 years old. The patients were randomly classified into two equal groups. The control group received usual medical care only, whereas the rehabilitation group received a selected home-based pulmonary rehabilitation exercise program plus the same usual medical care. The Physical Fitness Index (PFI), Chalder fatigue index, SF-36 questionnaire, dyspnea scale, and six-minute walk test (6 MWT) were measured before and after 12 weeks of intervention.

**Results:**

The rehabilitation group showed a significant lower mean of Chalder fatigue (11.1 ± 0.94) and a higher mean of 6MWT (439.7 ± 25.3) and PFI (52.3 ± 10.2), in addition to a higher mean of the SF-36 Questionnaire (66.4 ± 3.7) and a significant improvement of dyspnea in the mMRC score (26.7%), grade 2, (63.3%), grade 1 (10%), and grade 0 with a p-value < 0.001 when compared to the control group.

**Conclusion:**

Home-based pulmonary rehabilitation (HBPR) for patients with post COVID-19 syndrome is effective and has a potential direct influence on exercise capacity, fatigue, dyspnea, and quality of life. HBPR could be considered an adjunctive, applicable, and low-cost therapy for patients with post COVID-19 syndrome.

*Trial registration:* The study was registered in Pan African Clinical Trial Registry as a clinical trial ID (PACTR202111640499636), November 2021.

## Introduction

Coronavirus 2019 (COVID-19) is an epidemic condition that has affected more than 510 million people and caused death for more than 6,2 million people around the world [1]. COVID-19 causes various symptoms that affect the cardiovascular, respiratory, renal, neurological, and gastrointestinal systems of the body [2, 3].

COVID-19 has recorded a high rate of hospitalization and mortality; nearly 33% of hospitalized patients reported extreme loss in mental function, dyspnea, fatigue, reduced lung function, lessened exercise fitness, and poor quality of life [4]. Some people who were infected with the COVID-19 virus suffered long-term complications known as long COVID-19 or Post COVID-19 syndrome. This could be identified at the fourth week after infection. The most common symptoms of post-COVID syndrome are tiredness, fatigue, dyspnea, and depression. These symptoms may last weeks, months, or even years after the infection [5, 6].

Recent investigations concluded that 70% of post COVID-19 patients remain up to 12 months after hospital discharge with a 6-min walking distance (6MWD) below the estimated values [7]. Further studies stated a significant reduction of 6MWD in post COVID-19 patients when compared to healthy subjects, the prevalence of reduction reached up to 33% lower than the predicted values for a follow-up time of 2–6 months after discharge [8, 9]. Whereas Polese et al., observed that 6MWD was less than 75% of predicted values in a significant number of post COVID-19 patients. As COVID-19 affects multisystem such as the respiratory, cardiovascular, neurological, and musculoskeletal systems, reduction of 6MWD may be related to fatigue, dysnea, exertional hypoxemia, impairment of pulmonary microvasculature, and marked loss of muscle strength after hospital discharge [7, 10–12].

So, rehabilitation after discharge was raised as a vital treatment component. Pulmonary rehabilitation (PR) has a vital role in dealing with patients suffering from COVID-19 and its consequences, helping patients with post COVID-19 syndrome to be as independent as possible [2, 3, 13].

Post-discharge pulmonary rehabilitation should be applied as early as possible with a suitable evaluation of different symptoms like ease of breathing and cardiovascular endurance [14]. Pulmonary rehabilitation (PR) is an international rehabilitation method that consists of physical training for the musculoskeletal system and psychological and social education. PR was conducted on patients suffering from chronic respiratory diseases like chronic obstructive pulmonary disease (COPD) to decrease psychological problems such as depression, increase respiration capacity, and improve quality of life [15]. Pulmonary rehabilitation components include limb strengthening, endurance training as well as diaphragmatic and respiratory muscles training [16].

As we know, social separation is one of the most effective prevention methods for hindering the spread of COVID-19 in new cases [17]. Different communication tools were developed to avoid direct contact, such as video conferences, recorded videos, telephone assessments, online sessions, and tele-rehabilitation. These methods were used to treat pulmonary, skeletal, neurological, and cardiac conditions [18–20].

A model that enables access to pulmonary rehabilitation is known as home-based pulmonary rehabilitation (HBPR). It could be considered to avoid direct contact and to be delivered with minimal resources. HBPR has equivalent outcomes to center-based programs. Compared to usual care, HBPR is effective in improving functional exercise capacity. The home-based pulmonary rehabilitation program (HBPR) originated with adequate assessment for diagnosis and a teaching program with motivational interviews [21–23].

Barman et al. [24] recommended that we need clinical studies to explain the role of respiratory rehabilitation maneuvers on patients’ functional performance, ability, and quality of life post-acute respiratory syndrome and post COVID-19. Important issues raised during the pandemic were to use minimal resources, apply less direct personal contact, and at the same time get maximum treatment benefits. So, this study was conducted to investigate the influence of home-based pulmonary rehabilitation on patients with post COVID-19 syndrome through home program exercises.

## Methods

### Study design

A randomized controlled trial was implemented in the outpatient clinic, Faculty of Medicine, Beni-Suef University, between December 2021 and May 2022. The Committee of Research Ethics, Faculty of Medicine, Beni-Suef University (FM-BSU-REC) authorized this work from the ethical point of view (No. FMBSUREC/07092021/Alsharawy). The protocol was registered at the Pan African Clinical Trial Registry (PACTR202111640499636) as a clinical trial.

### Participants

Seventy-five patients with post COVID-19 syndrome (42 male and 33 female) were selected randomly. Their ages extended from 40 to 70 years old. All the selected patients followed the study inclusion criteria, which were: diagnosed and confirmed via COVID-19 polymerase chain reaction (PCR) test within the last three months, either hospitalized or receiving home treatment but not needing ICU admission; discharged at least one month of acute phase recovery with post COVID-19 fatigue, dyspnea, and exercise intolerance; assessment of dyspnea by mMRC ranged from 2 to 3; patients scores on the Chalder fatigue scale ranged from 14 to 23 [25] patients were medically stable and non-smokers. All patients were allocated to the outpatient clinic, Faculty of Medicine, Beni-Suef University. The aim of the study was to educate the entire participant. The patients signed a consent form for the participation agreement.

Patients experiencing chronic lung disease, severe cardiovascular disease, diabetes, advanced musculoskeletal disorders, cancer, acute and chronic kidney disease, and needing oxygen support therapy were excluded from the study. Also, those who received any medication interfered with muscle power. Patients who were unable to walk were also excluded. There are no orthopedic or neurological restrictions.

### Randomization

**Fig. 1:**
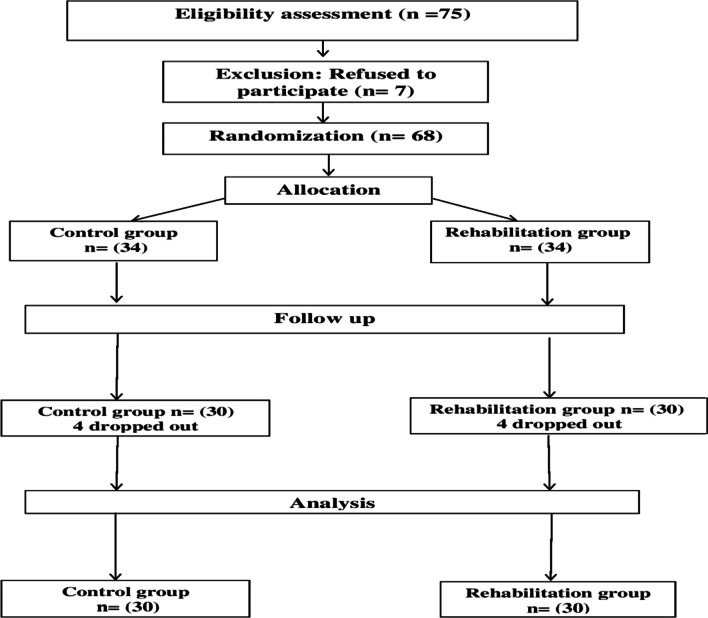
Flow chart diagram

Seventy-five patients with post-COVID-19 syndrome were evaluated for eligibility; seven patients disagreed with participating in this study. A physical therapist blinded to hypothesis allocated 68 patients randomly into two groups of equal size by using (Graphpad software, Inc.) [26], through opening sealed randomization block envelopes (blocks of 5) in each group to minimize selection bias. Eight patients (four in each group) dropped out for follow-up assessments due to non-specific reasons, so the analysis of the data is applied to 60 patients, with 30 in each group, as shown in Fig. 1.

The control group received no exercise program plus their usual medical care which consisted of multivitamins, antioxidant, healthy high protein diet and healthy life style, whereas any patient who received any medication that interfered with muscle power was excluded. The rehabilitation (Rehab) group received the selected home-based pulmonary rehabilitation exercise program plus the same medical care. Interventions lasted up to twelve weeks.

### Therapeutic procedure for rehabilitation group

Firstly, the patient’s interview was conducted by the instructor to explain, teach, and perform exercise models and educate them on exercise instructions. All patients in the rehabilitation (rehab) group were instructed to perform daily home exercises for three months. Tele-monitoring was conducted and regulated on a weekly basis to follow patient adherence to exercise. For patient follow-up and reassessment, face-to-face visits were regulated after 2, 4, 8, and 12 weeks of patient adherence to the exercise program [2, 27]. The rehabilitation exercise program was as follows:

- Regular walking for 30–60 min, 5 days a week, at a normal pace for 3 months
- Respiratory muscle training (diaphragmatic strengthening) for 10–15 min, twice per day, daily for 3 months. Diaphragmatic strengthening was done by using a minimal weight of 1–2 kg for all patients placed on the abdomen and patients lying supine at 45 degrees. A belt was tightened around the patient’s abdomen, and the weight was placed in a pocket of this belt. The belt was worn in a manner that seemed neither loose nor firm.
- Resisted training 3 sets of 10 repetitions, twice a day, 5–7 days a week, using a low weight of 1–3 kg for both quadriceps and selected upper limb muscle groups (shoulder abduction, horizontal abduction, and elevation) for 3 months.

### Outcome measurements

*1. 6-min walk test (6MWT):* a measurement tool used to assess patient endurance and exercise capacity. 6MWT is a submaximal test used to reflect the patient's ability to perform activities of daily living [28]. Before and after the treatment interventions, 6MWT evaluated every patient in each group**.**

*2. Harvard Step Test (Physical Fitness Index) (PFI):* A measurement test for muscular work and its recovery was used mainly to reflect cardiopulmonary efficiency. Patients were asked to step up and down on a step of 20 and 18 inches for males and females, respectively. The patients performed the test as much as they tolerated, but not more than 5 min, and each patient's time was recorded. After test completion, the heart rate recovery numbers 1, 2, and 3 were recorded [29]. PFI was calculated by the following equation:

$${\text{PFI}} = \frac{{{\text{test}}\;{\text{duration}}\;{\text{in}}\;{\text{second}} \times 100}}{{{\text{Heart}}\;{\text{rate}}\;1 + 2 + 3}}$$

PFI was carried out for the patients in the two groups before and after treatment interventions.

*3. Short Form 36 Health Survey Questionnaire (SF-36):* an overall indicator for health status involving eight items reflecting patients’ general health from physical and social aspects. SF-36 was introduced to all participants in pre- and post-treatment interventions. A valid and reliable Arabic version of SF-36 was used in the two groups before and after treatment interventions [30].

*4. The Chalder Fatigue Scale (CFQ11):* a questionnaire that provides a brief objective tool for exploring the severity of physical and mental fatigue. It’s now widely used in academic and applied occupational medicine. CFQ11 consisted of 11 items; seven represented physical fatigue and four were psychological fatigue. The Likert scoring method was used to express responses from 0 as no symptoms to 3 as severe symptoms (Craig, 2015). CFQ11 was introduced to all participants during pre- and post-treatment interventions.

*5. The Modified Medical Research Council Dyspnea Scale (mMRC):* it was used to assess a patient's dyspnea level (shortness of breath). The mMRC grading scale scores range from 0 to 4, with 0 indicating breathless during heavy exercise. However, score 4 implies breathless when dressing [31]. All patients were assessed for dyspnea before and after the intervention.

### Power analysis

Calculating sample size was done through version 3.1.7 of G-Power^©^ software (Experimental Psychology Institute, Heinrich Heine University, Germany). Thirty patients in each group were the minimum sample size. Depending on previous research results with an effect size of 1.32, Two-sided (two tails) type I error of 0.05 and power of 95%.

### Statistical analysis

Version 22 of SPSS software (SPSS Inc., USA) was used to manipulate the collected data. All the data were normally distributed and so all the tests were parametric. A t-test compared quantitative measures between two independent groups. A paired t-test was used to compare two dependent quantitative data. The chi-square test had been used to compare qualitative data. Correlations between variables were tested by a bivariate Pearson correlation test. The P-value < 0.05 was considered statistically significant.

## Results

Table 1 illustrates that there was no statistically significant difference (p-value > 0.05) between rehabilitation and control groups in age, anthropometric measures, or demographic data.

**Table 1:** Comparisons of demographic and anthropometric characteristics of rehabilitation and control groups

Variables	Rehabilitation (N = 30)	Control (N = 30)	P-value
	Mean ± SD	Mean ± SD	
Age (years)	56.9 ± 6.7	55.5 ± 7.1	0.4^**(T)^
Height (cm)	170.2 ± 3.8	168.3 ± 5.1	0.1^**(T)^
Weight (kg)	87.5 ± 4.3	85.2 ± 5.8	0.09^**(T)^
BMI (kg/m^2^)	30.1 ± 0.83	29.1 ± 4.4	0.2^**(T)^

Sex	N. (%)	N. (%)	
Male	16 (53.3%)	17 (56.7%)	0.9^**(C)^
Female	14 (46.7%)	13 (43.3%)	

**Nonsignificant, *T* t-test, *C*  chi-square test, *N*  number

Table 2 explained that there was no statistically significant difference (p-value > 0.05) between rehabilitation and control groups regarding the baseline clinical assessment of Chalder fatigue, 6MWT, the Physical Fitness Index (PFI), the SF36 Questionnaire, and dyspnea degrees.

**Table 2:** Comparison of clinical assessment before intervention between rehabilitation and control groups

Variables	Rehabilitation (N = 30)	Control (N = 30)	P-value
	Mean ± SD	Mean ± SD	
Chalder fatigue	19.3 ± 2.3	18.9 ± 2.5	0.5^**(T)^
6MWT	270.7 ± 34.7	274.1 ± 31.8	0.7^**(T)^
PFI	17.7 ± 3.7	17.2 ± 3.5	0.6^**(T)^
*SF-36 Questionnaire*			
Total score	16.4 ± 1.7	17.6 ± 3.4	0.07^**(T)^

mMRC	N. (%)	N. (%)	P-value
Moderate	9 (30%)	8 (26.7%)	0.9^**(C)^
Sever	21 (70%)	22 (73.3%)	

**Nonsignificant, *T* t-test, *C*  chi-square test, *N*  number

Comparison of pre- and post-treatment values for each group shows a statistically significant decrease in Chalder fatigue, a significant increase in 6MWT, PFI, and SF-36 questionnaires, and a higher percentage of improvement in the degree of dyspnea score (mMRC) with a p-value < 0.001 (Table 3).

**Table 3:** Clinical assessment before and after intervention in each group

Variables	Rehabilitation (n = 30)		P-value	Control (n = 30)		P-value
	Pre	Post		Pre	Post	
	Mean ± SD	Mean ± SD		Mean ± SD	Mean ± SD	
Chalder fatigue	19.3 ± 2.3	11.1 ± 0.94	< 0.001*^(PT)^	18.9 ± 2.5	13.4 ± 2.1	< 0.001*^(PT)^
6MWT	270.7 ± 34.7	439.7 ± 25.3	< 0.001*^(PT)^	274.1 ± 31.8	347 ± 32.7	< 0.001*^(PT)^
PFI	17.7 ± 3.7	52.3 ± 10.3	< 0.001*^(PT)^	17.2 ± 3.5	36.7 ± 4.2	< 0.001*^(PT)^
*SF-36 Questionnaire*						
Total score	16.4 ± 1.7	66.4 ± 3.7	< 0.001*^(PT)^	17.6 ± 3.4 ±	47.6 ± 2.6 ±	< 0.001*^(PT)^

mMRC	N. (%)	N. (%)	P-value	N. (%)	N. (%)	P-value
No (0)	–	3 (10%)	< 0.001*^(C)^	–	0 (0%)	< 0.001*^(C)^
Mild (1)	–	19 (63.3%)		–	11 (36.7%)	
Moderate (2)	9 (30%)	8 (26.7%)		8 (26.7%)	19 (63.3%)	
Severe (3)	21 (70%)	–		22 (73.3%)	–	

*Significant, *PT* paired t-test, *C*  chi-square test, *N*  number

**Fig. 2:**
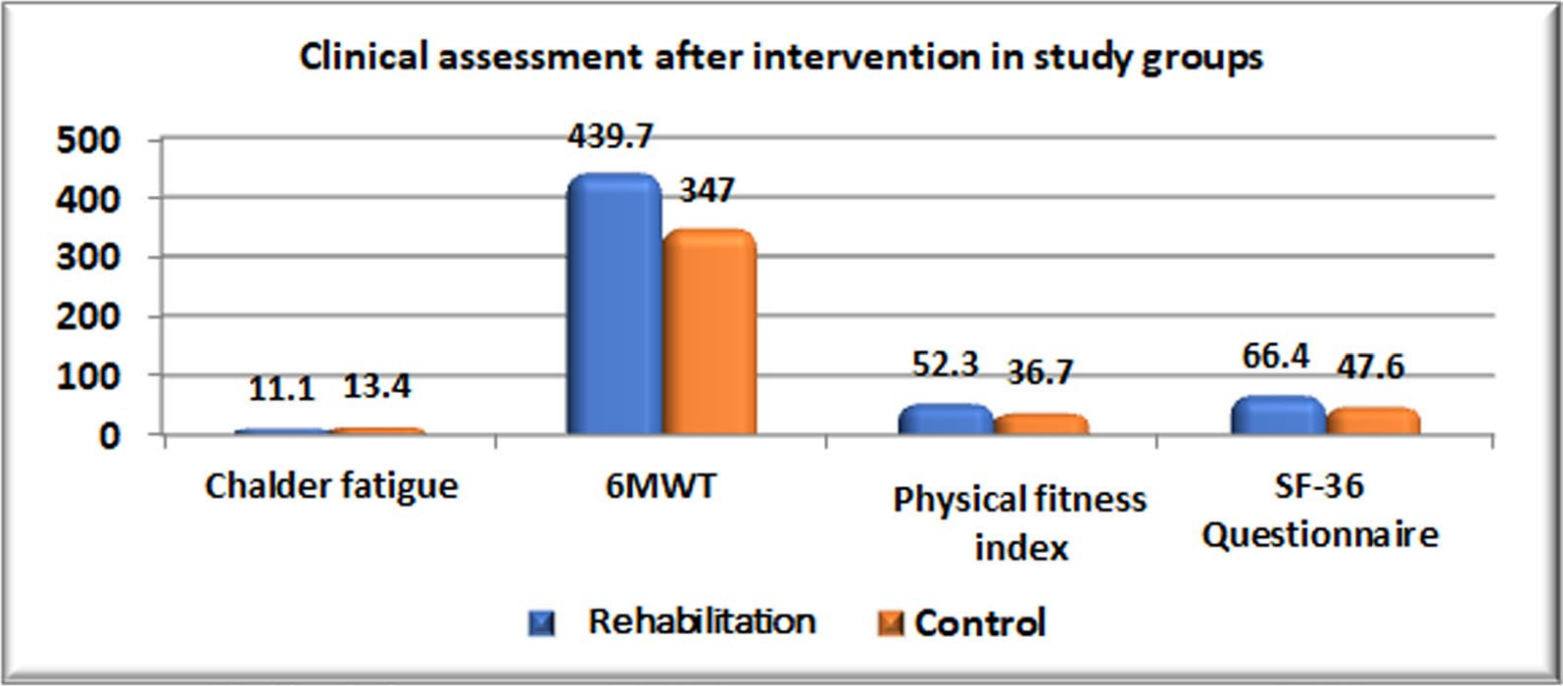
Clinical assessment after intervention in study groups

**Fig. 3:**
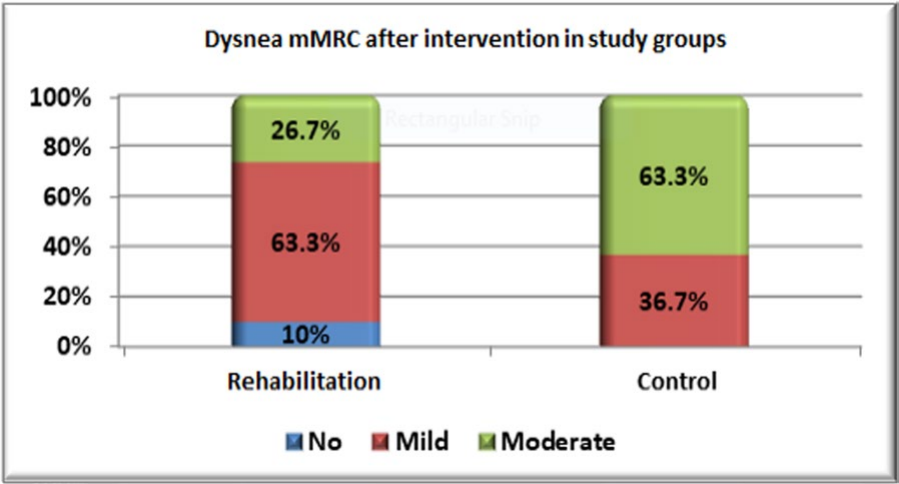
Degree of dyspnea score (mMRC) after intervention in study groups

After implementation of intervention, comparison of rehabilitation group to control group stated that rehabilitation group showed much significant lower mean of Chalder fatigue, higher mean of 6MWT, PFI, and SF-36 Questionnaire with p-value < 0.001 in addition to higher percentage of improvement in degree of dyspnea score (mMRC) with p-value < 0.008 as described in (Table 4) and (Figs. 2 and 3).

**Table 4:** Comparison of clinical assessment after intervention between rehabilitation and control groups

Variables	Rehabilitation (N = 30)	Control (N = 30)	P-value
	Mean ± SD	Mean ± SD	
Chalder fatigue	11.1 ± 0.94	13.4 ± 2.1	< 0.001*^(T)^
6MWT	439.7 ± 25.3	347 ± 32.7	< 0.001*^(T)^
PFI	52.3 ± 10.3	36.7 ± 4.2	< 0.001*^(T)^
*SF-36 Questionnaire*	66.4 ± 3.7	47.6 ± 2.6	< 0.001*^(T)^

mMRC	N. (%)	N. (%)	P-value
No	3 (10%)	0 (0%)	0.008*^(C)^
Mild	19 (63.3%)	11 (36.7%)	
Moderate	8 (26.7%)	19 (63.3%)	

*Significant; p< 0.05, *T* t-test, *C*  chi-square test, *N*  number

The next table showed a statistically significant negative correlation with a p-value < 0.05 between BMI and total SF-36 questionnaire score before intervention; in addition, there was a statistically significant positive correlation with a p-value < 0.05 between dysnea mMRC score after intervention with both age and BMI. However, there was no statistically significant correlation with a p-value > 0.05 between other clinical assessment measures and age or BMI (Table 5).

**Table 5:** Correlation between age and BMI with clinical assessment among study groups before and after intervention

Variables	Age (years)		BMI (kg/m^2^)	
	r	P-value	r	P-value
*Pre intervention*				
mMRC	0.13	0.3^(R)^	0.05	0.7^(R)^
Chalder fatigue	0.05	0.7^(R)^	0.05	0.7^(R)^
6MWT	− 0.11	0.4^(R)^	− 0.01	0.9^(R)^
PFI	− 0.01	0.9^(R)^	− 0.12	0.4^(R)^
Total SF-36 Questionnaire score	− 0.17	0.2^(R)^	− **0.29**	0.02*^(R)^
*Post intervention*				
mMRC	**0.27**	0.03*^(R)^	**0.26**	0.04*^(R)^
Chalder fatigue	− 0.01	0.9^(R)^	0.1	0.4^(R)^
6MWT	0.05	0.7^(R)^	0.14	0.3^(R)^
PFI	0.08	0.6^(R)^	0.12	0.3^(R)^
Total SF-36 Questionnaire score	0.12	0.4^(R)^	0.18	0.2^(R)^

*Significant; p<0.05, *R* Pearson correlation test

## Discussion

The present research paper investigated the impact of home-based pulmonary rehabilitation for post COVID-19 patients through constructed and supervised home program exercises. It was established that an exercise program improves general health and extremity strength compared with no exercise. The current study compared two groups of post COVID-19 syndrome patients: the control group, who received usual medical care only, and the rehabilitation group, who received a pulmonary rehabilitation program conducted at home. The results of this study revealed an improvement in both groups in the 6MWT, the PFI, the Chalder fatigue index, and the SF-36 questionnaire, with a favor for the rehabilitation group.

Our research results concurred with the results of the Gonzales et al. [32], Rodríguez et al. [33] studies, which accepted that tele-rehabilitation and home-based rehabilitation improved lung function when assessing 6MWT. Gonzales et al. [32] established that tele-rehabilitation through 10 modified breathing exercise protocols for a week in the acute condition of COVID-19 improved the exercise capacity assessed by 6MWT in the study group compared to the control group. Rodríguez et al. [29] disclosed that using breathing exercise or strengthening exercises for 2 weeks significantly improved the dysnea and physical state of COVID-19 patients when compared to no intervention on the evaluation of a 6-min walk test in favor of breathing exercises.

Furthermore, the rehabilitation group of the current study performed diaphragmatic strengthening exercises twice per day for 3 months and walked 5 days a week as an aerobic exercise. The rehabilitation group's results were similar to those of Amaral et al. [34] who looked at COVID-19 survivors who did a twelve-week guided home training program that included nine different resistance exercises for all body parts and aerobic exercise like walking two and five times a week compared to those who did nothing. Additionally, Li et al. [35] investigated tele-supervised home-based exercises consisting of breathing exercises, chest expansion exercises, aerobic exercises, and lower limb strengthening exercises with 40–60-min sessions, 3 times per week for 1.5 months, compared with control for COVID-19 survivors. Their results showed that there was a significant difference in the 6-min walk test, extremity muscle power, and quality of life in favor of the rehabilitation group. In agreement with our result, Cancino et al. [36] explored COVID-19 patients who performed 2–3 sessions per week at home under a remote rehabilitation program. Each session lasted up to an hour and was composed of aerobic and resistance training. At the end of the 24th session, there was a highly significant improvement in general health status in the form of increased aerobic fitness, indicating that a home rehabilitation program would return persons post COVID-19 quickly to normal life.

Likewise, Martin et al. [37] used videoconferencing as a tele-rehabilitation method to convey pulmonary rehabilitation via an experienced practitioner for patients who achieved home-based exercises twice a week for 1.5 months, each session composed of ½ hour of endurance exercises and upper and lower extremity resistance training for 1/3 h. The results of this study revealed momentous enhancements in functional exercise capacity, approving the feasibility and validity of a tele-rehabilitation regimen for COVID-19 patients’ recovery**.**

There are few studies to examine the efficacy of tele-rehabilitation and home-based physical therapy programs for COVID-19 patients. All of these studies showed that the improvement in the rehabilitated group was above the control group; this matched our result in the current study [38].

A systematic review of pulmonary rehabilitation was conducted on patients who had long-term effects of COVID-19 after recovery. Pulmonary rehabilitation was delivered through different modes, such as tele-rehabilitation and direct contact PR. The results of this review come in accordance with our study, as it proved that a PR program is better than no intervention in improving fatigue and functional capacity in patients with COVID-19. This also assured safety in the management of different severities of COVID-19 [39].

Regarding quality of life (QOL), the above-mentioned systematic review stated that tele-rehabilitation didn’t prove significant improvement compared to no rehabilitation; otherwise, face-to-face PR proved significant improvement. This result contradicts our finding of QOL, which may be related to differences in participants' demographic and clinical features, as well as the stage and severity of the disease.

A reduction in dyspnea perception during exercise training, improved exercise capacity, and fatigue might be due to physiological adaptation to exercise training [40]. Improved measured outcomes such as exercise capacity, fatigue, and physical fitness index in the current study may be revealed to enhance lung compliance, improve alveolar ventilation and oxygenation, and improve respiratory and skeletal muscle conditioning [41].

Explanations for improvement may be related to the fact that our study followed the recommended international guidelines for maximum benefit of the pulmonary rehabilitation (PR) program, which was performed 3–5 times per week for 3 months with a minimum session time of 20 min. The pulmonary rehabilitation (PR) program is composed of endurance exercises, interval training, and strengthening exercises of the extremities in addition to walking [13].

Additionally, walking increases aerobic capacity, which boosts immunity and immunoglobulin levels, improves the respiratory system in a way similar to antibiotics, antioxidants, and antimycotics, keeps lung function, texture, and respiratory function up, and lowers psychological symptoms in COVID-19 patients [42]. Home-based rehabilitation has the advantages of preventing the risk of infection and recurrence of COVID-19 and encouraging patients to be independent and responsible for their health.

Our results also showed significant improvement in the control group (received usual medical care) when compared to baseline values at the end of the three months of study which may be related to slowly resolved symptoms overtime, subsidence of inflammatory mediators, improved eating habits and relieved stress [43], however, comparing these results to those of the rehabilitation group stated a significant difference with a favor for the rehabilitation group.

Our results also showed that there was a statistically significant negative correlation with a p-value < 0.05 between BMI and total SF-36 questionnaire score before intervention, and in addition, there was a statistically significant positive correlation with a p-value < 0.05 between dysnea mMRC score after intervention with both age and BMI. On the other hand, there was no statistically significant correlation with a p-value > 0.05 between other clinical assessment measures and age or BMI.

Likewise, Suraj et al. [44] showed that there was a weak positive correlation (r = 0.0385) p > 0.05, which is not of statistical significance, between the severity of dyspnea and age. Also, pre-exercise dyspnea measured by the mMRC scale in obese people significantly correlated with post-exercise dyspnea Borg scores, independent of the presence or absence of airflow obstruction. Ramanathan and Chandrasekaran [45] concluded that the 6 MWD correlated significantly (P < 0.05) with age, height, and BMI. The natural decline in muscle mass, strength, and maximal oxygen uptake that occurs with aging could be the reason why advancing age has a negative impact on the 6 MWD. Alicja et al. [46] showed that early rehabilitation programs appear to be critical for COVID-19 recovery**.**

Our study limitations may be due to participants’ variations in their mode of life or their willingness to follow exercise instructions, which could interfere with study results.

## Conclusion

Home-based pulmonary rehabilitation (HBPR) requires fewer resources and assures less patient contact as compared to center-based rehabilitation. HBPR has its own economic advantage and more safety measures. We need more studies on home-based rehabilitation methods for COVID-19, where COVID-19 is a relatively new condition, and studying the long-term effects of exercises on physical fitness and quality of life with these patients is required. Home-based pulmonary rehabilitation (HBPR) for patients with Post COVID-19 syndrome is effective, safe, and has a potential direct effect on exercise capacity, fatigue, dyspnea, and quality of life. HBPR could be considered an adjunctive, low-cost therapy for patients with post COVID-19 syndrome.

## Data Availability

The data associated with the paper are available from the corresponding author on reasonable request.

## Abbreviations

HBPR: Home based pulmonary rehabilitation
6MWT: 6-Min walk test
PFI: Physical Fitness Index
SF-36: Short Form 36 health survey Questionnaire
CFQ11: Chalder Fatigue Scale
mMRC: Modified Medical Research Council Dyspnea Scale
